# Plasma Membrane Remodeling During Microglial Activation: A Hypothesis Linking Microglial Shape and Lipid Droplet Formation

**DOI:** 10.1002/glia.70193

**Published:** 2026-06-25

**Authors:** G. William Rebeck, Giorgi Shautidze, Jordy Sepulveda, Gracie S. Healey, Priyanka S. Narayan

**Affiliations:** ^1^ Department of Neuroscience Georgetown University Washington District of Columbia USA; ^2^ Department of Pharmacology & Physiology Georgetown University Washington District of Columbia USA; ^3^ Center for Neuroimmunology and Glial Biology Institute of Molecular Medicine, University of Texas Health Science Center Houston Texas USA; ^4^ Genetics and Biochemistry Branch National Institute of Diabetes and Digestive and Kidney Diseases, National Institutes of Health Bethesda Maryland USA

**Keywords:** fatty acids, lipid droplets, microglia, morphology, motility, plasma membrane, triacylglycerols

## Abstract

Microglia are dynamic cells that respond both transcriptionally and morphologically to acute brain injury as well as to chronic neurodegenerative conditions. Upon activation, they become less ramified, more rounded, and accumulate intracellular lipid droplets. In this hypothesis paper, we propose that the formation of these lipid droplets supports the redistribution of plasma membrane lipids required during morphological remodeling. We rely on original and published studies of microglial morphology under conditions of aging, acute activation, and chronic activation. In ex vivo brain slices, microglia responded to either ATP or acute Aβ injections within minutes by extending their proximal processes toward the stimulus while simultaneously retracting their distal processes into their cell bodies. Chronic exposure to Aβ in mouse models of amyloid reduced microglial branching alongside a two‐ to three‐fold loss of surface area. Transcriptomic analyses showed that activated microglia upregulate genes involved in fatty acid synthesis and fatty acid activation, both processes that are necessary in the production of triacylglycerol. Integrating these new and published analyses of microglia, we developed a hypothesis in which plasma membrane phospholipids are redistributed during acute activation and, during chronic activation, they are metabolized to triacylglycerol into lipid droplets. Tests of this hypothesis, through various pharmacological and genetic approaches, would contribute to our understanding of lipid droplets in cells that undergo substantial morphological changes.

## Graphical abstract

Microglial morphology changes in response to damage by Aβ and aging result in significant reductions in cell surface area. Transcriptomic analyses suggest that fatty acid synthesis and triacylglycerol formation drive redistribution of surface membrane into intracellular lipid droplets.

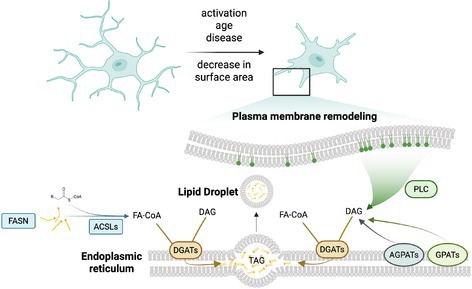

## 1 Introduction

Several genetic risk factors for late onset Alzheimer's Disease (AD) have functions related to lipid metabolism and lipid trafficking (Jones et al. 2010; Kunkle et al. 2019). Of these genetic factors, the most impactful are the alleles of the *APOE* gene (Strittmatter et al. 1993). *APOE* encodes apolipoprotein E (apoE), a lipid transport protein that is the most abundant CSF apolipoprotein (Husain et al. 2021) and is highly expressed in glia (Pitas et al. 1987). The *APOE4* allele confers increased AD risk while *APOE2* and rare alleles (Chen et al. 2021) such as Christchurch (Quiroz et al. 2024) and Jacksonville (Liu et al. 2021) are protective. Disruptions to glial lipid metabolism were originally described as one of the hallmarks of AD (Alzheimer et al. 1995) and recent studies have centered on disruptions to lipid biology in AD risk, pathogenesis, and progression (Blanchard et al. 2022; Green et al. 2024; Haney et al. 2024; Lee et al. 2023, 2021; Sienski et al. 2021; Stephenson et al. 2025; Victor et al. 2022; Yang et al. 2023). In the CNS, apoE affects many aspects of lipid metabolism, including lipid uptake, lipid efflux, and lipid storage (Jackson et al. 2024). Lipid storage can occur in the form of lipid droplets (LD), organelles composed of a phospholipid lipid monolayer surrounding a hydrophobic neutral lipid core (Ralhan et al. 2021). LDs affect lipid storage (Farmer et al. 2019), energy production (Kumar et al. 2025), or lipid transport to other cells (Ioannou et al. 2019; Qi et al. 2021) and likely serve different functions in different cell types. The *APOE4* genotype is associated with higher levels of LD in microglia in culture (Farmer et al. 2019; Sienski et al. 2021) and in human brains (Haney et al. 2024).

Microglia perform many functions that are relevant to the neurodegeneration seen in AD: promoting inflammatory processes, phagocytosis of myelin debris and apoptotic cells, and monitoring and pruning of synapses (Colonna and Butovsky 2017; McKinney et al. 2026). Microglia are also relevant to AD based on AD genetic risk studies that identified several genes expressed mainly in microglia (Romero‐Molina et al. 2022). Additionally, RNA sequence analysis of microglia in brains and in cell culture has identified many transcripts related to lipid immunoregulation and lipid metabolism after activation seen in neurodegeneration (Haney et al. 2024; Lee et al. 2023; Marschallinger et al. 2020; Stephenson et al. 2025). These genetic and transcriptomic associations imply that microglial lipid metabolism could affect the onset or progression of AD (Sudwarts and Thinakaran 2023).

LD accumulate in microglia that undergo activation with aging and AD (Haney et al. 2024; Marschallinger et al. 2020), conditions that are associated with dramatic changes in microglial shape. Under homeostatic conditions, microglia have small cell bodies and extensive arbors of finely branched processes that enable efficient surveillance of the CNS environment (Ransohoff and Perry 2009). After detection of signs of damage (such as ATP, laser ablations, or protein accumulations), microglia have immediate extension of some processes toward the signal (Bolmont et al. 2008; Davalos et al. 2005; Franco‐Bocanegra et al. 2019; Gyoneva et al. 2016; Liu et al. 2023; Sepulveda et al. 2024). Over a more chronic time frame, activated cells progressively become more rounded, with a larger cell body and fewer, shorter processes (Vidal‐Itriago et al. 2022). These indications of activation have also been observed in normal aging. In human brain tissue samples, microglia display reduced arborization and fragmented processes (Davies et al. 2017; Streit et al. 2004); and positron emission tomography with PK11195 showed evidence of microglial activation with aging (Schuitemaker et al. 2012). Reductions in highly ramified microglia have been found in numerous animal models, with increases in the number of activated, dystrophic, and amoeboid microglia with large cell bodies, including: rat (Myers et al. 2026); mice (Choi et al. 2022; Henze et al. 2026); sheep (Carr et al. 2026); and marmosets (Phillips et al. 2026; Rodriguez‐Callejas et al. 2019).

Studies of microglial activation in real time are enabled by techniques that label microglia with fluorescent markers. One model to allow appreciation of microglial changes in vivo is a system where a fluorescent marker is expressed in microglia, such as the CX3CR1‐driven expression of green fluorescent protein (GFP) (Jung et al. 2000). In experiments here, we examined acute changes in microglial shapes during responses to CNS damage and paired our findings with existing transcriptomic and lipidomic datasets to analyze how changes in lipid metabolism relate to concurrent changes in cell shape. From these analyses of published and original research, we develop a novel hypothesis that there is a direct biochemical relationship between the LD accumulation and membrane remodeling that both occur with acute and chronic changes to microglial shape.

## 2 Methods

### 2.1 Mice

CX3CR1^GFP/GFP^ mice (JAX stock No. 005582) on a C57BL/6J background (JAX stock No. 000664) (Sepulveda‐Rodriguez et al. 2019) were crossed with *APOE3KI* (JAX stock No. 029018) or *APOE4KI* (JAX stock No. 027894) (Foley et al. 2022) mice to obtain *APOE3* ^*+/+*^, CX3CR1^GFP/−^ and *APOE4* ^*+/+*^, CX3CR1^GFP/−^ mice (Sepulveda et al. 2024). All animals were housed with littermates and kept on a 12‐h light/dark cycle with *ad libitum* access to chow and water. Male and female *APOE3* ^*+/+*^, CX3CR1^GFP/−^, and *APOE4* ^*+/+*^, CX3CR1^GFP/−^ mice were euthanized at four to six months of age (*n* = 4–5 mice per genotype/sex). In addition, *APOE4* ^*+/+*^, CX3CR1^GFP/−^ mice were crossed with 5XFAD mice on a C57BL/6J background (JAX stock No. 034848) to generate *APOE4* ^*+/+*^, CX3CR1^GFP/−^, 5XFAD^+/−^ mice (*n* = 4 mice).

All studies were carried out following the Guide for the Care and Use of Laboratory Animals as adopted by the U.S. National Institute of Health and approved by Georgetown University Animal Care and Use Committee, approval protocol 2016‐1160.

### 2.2 Microglial Morphology Changes in Response to Biochemical Signals

Microglia motility data was collected as part of a study on process motility related to *APOE* genotype and age (Sepulveda et al. 2024). Mice were anesthetized with unmetered isoflurane and intracardially perfused with NMDG solution containing N‐methyl‐D‐glucamine (NMDG) 92 mM, KCl 2.5 mM, NaH_2_PO_4_ dihydrate 1.25 mM, NaHCO_3_ 30 mM, HEPES 20 mM, glucose 25 mM, sucrose 10 mM, ascorbic acid 5 mM, thiourea 2 mM, sodium pyruvate 3 mM, N‐acetyl‐L‐cysteine 5 mM, MgSO_4_ heptahydrate 10 mM, and CaCl_2_ dihydrate 0.5 mM at pH 7.3–7.4 and osmolarity 300–310 mΩ/kg. For ex vivo studies, 300 μm horizontal slices were cut in ice‐cold NMDG using the Vibratome 3000 plus Sectioning System. Sections were incubated for 5 min in NMDG at 32°C, followed by recovery for 30 min at 32°C in artificial cerebrospinal fluid (aCSF) containing NaCl 120 mM, KCl 3.5 mM, NaH_2_PO_4_ 1.25 mM, NaHCO_3_ 26 mM, CaCl_2_ dihydrate 1 mM, MgCl_2_ 7 mM, and dextrose 10 mM at pH 7.3–7.4 and osmolarity 300–310 mΩ/kg. Slices were transferred to room temperature (22°C–24°C) and equilibrated for greater than 10 min before use. All slices were used within 5 h of euthanasia, and all microglia studied had soma at least 30 μm from the cut surface. All experiments were performed in recording aCSF solution containing NaCl 124 mM, KCl 3.5 mM, NaH_2_PO_4_ dihydrate 1.2 mM, NaHCO_3_ 26 mM, CaCl_2_ dihydrate 2 mM, MgCl_2_ 1 mM, and dextrose 10 mM at pH 7.3–7.4 and osmolarity 300–310 mΩ/kg. Recording aCSF solution was maintained at pH 7.4 by bubbling with carbogen gas (95% O_2_/5% CO_2_, Roberts Oxygen).

For ATP effects on microglia morphology, a patch pipette containing 3 mM ATP in aCSF was lowered into the ex vivo brain tissue and the ATP was allowed to diffuse into the tissue without pressure in the pipette. During active process motility, 1024 × 1024 pixel ZT‐stack images from 11 planes (1.5 μm apart) were taken periodically over 30 min. When necessary, time lapses were stabilized using the StackReg plugin (Thevenaz et al. 1998) in Image J (Schindelin et al. 2012). For Aβ responses, HiLyte Fluor 555‐labeled Aβ42 (1 mg/mL in 1% NH_4_OH) was diluted in aCSF and up to 5 μL was injected into brain tissue. ZT‐stack images from 20 planes (0.5 μm apart) were acquired in separate channels every 15 min for 2 h. Time‐lapse images were manually thresholded, binarized, and color‐coded (green pixels = microglia, red pixels = Aβ42) in Image J.

### 2.3 Surface Area Analysis

All mice for analysis of cell surface area were treated with a control intravenous injection of an IgG1 antibody (Syd Labs) as part of a separate study. Mice were euthanized at 4 months of age; brains were perfused with cold PBS and fixed in paraformaldehyde. Hemi‐sectioned brains were dehydrated with three consecutive sucrose incubations at 10%, 20%, and 30% sucrose, frozen, and sliced at 30 μm. Slices were immunostained for Aβ using the mouse MOAB‐2 primary antibody (Novus). Confocal images were collected using a Leica Mica confocal microscope. “Lightning grade” resolution Z‐stacks with 0.19–0.24 μm z‐step size were obtained at 63× zoom with water immersion. Microglia were imaged at 499 nm excitation/519 nm emission (green) and plaque at 343 nm excitation/441 nm emission (blue).

Morphological reconstructions were performed using Imaris 10.2 software. Surface area and volume measurements were performed using the “machine learning” option of the surface map, with a surface detail of 0.3 μm. The surface area to volume ratio was used in analyses to control for occasional overlapping microglia or actively dividing microglia. Skeletal reconstruction was performed using the “filament” option, tracing the processes that were enveloped by the surface map. A total of 25 cells were analyzed, with 13 near to amyloid plaques (within 10 μm) and 12 far from amyloid plaques (beyond 100 μm). A measure of microglial complexity, the Branching index, was defined using the intersections of reconstructed line with circles drawn at 1 μm intervals (Garcia‐Segura and Perez‐Marquez 2014):

$$\begin{array}{c} \text{Branching Index}=\\ \Sigma \left(\text{Intersections}\;{\text{circle}}_{n}–{\text{Intersections circle}}_{n-1}\right)\times {\text{radius}}_{n} \end{array}$$

Analysis of microglia was done in a blinded manner based only on images of microglia without information about amyloid staining. Measures of individual microglia were compared using linear regression analysis and grouped data were compared using unpaired *t*‐tests.

### 2.4 Transcriptomic Analysis

All data used for transcriptomic analyses were generated as part of prior studies (Lee et al. 2023; Stephenson et al. 2025; Sun et al. 2023). Four separate analyses were performed. First, functional enrichment analyses using Metascape (Zhou et al. 2019) were performed on genes differentially expressed upon LPS treatment in microglia (Stephenson et al. 2025) (Tables [Link], [Link]). Second, lipid metabolism genes related to membrane and storage processes were compiled from KEGG pathways and prior literature (Mathiowetz and Olzmann 2024; Olzmann and Carvalho 2019) (Table S4). Published transcriptomic datasets were analyzed for changes in this gene list following LPS or Aβ treatments. Only statistically significant changes were used for human iPSC‐derived microglia (Stephenson et al. 2025; Sun et al. 2023) and concordant fold‐change direction was required for mouse data (Table S5) (Lee et al. 2023). Third, the differentially expressed genes in iPSC‐derived microglia following diacylglycerol acyltransferase (DGAT) inhibitor treatment were identified using previously published data (Stephenson et al. 2025) and functional enrichment was performed using Metascape (Zhou et al. 2019) (Tables S6 and S7). Finally, using a published dataset (Marschallinger et al. 2020), a list of 621 lipid‐droplet accumulating microglia (LDAM) genes were collated by identifying genes that were differentially expressed (adjusted *p*‐value ≤ 0.05) between LD “hi” and LD “lo” microglia in aged mice. Using Ensembl Biomart, human orthologues were identified for 501 of these genes and their fold changed was probed in transcriptomic datasets on iPSC‐derived microglia treated with LPS (Tables S1 and S8). Functional enrichment for differentially expressed genes in this list was also performed with Metascape (Zhou et al. 2019). For all functional enrichment analyses, summary terms shown are in the figure and full results in Supporting Information Tables.

### 2.5 Lipidomic Analysis

Previously published lipidomics data (Prakash et al. 2025) from mouse primary microglia treated with Aβ for 24 h were used to assess the effects of activation on lipid species. In this analysis, the fold change of abundance of each lipid was plotted and separated by lipid class.

## 3 Results

### 3.1 Acute Microglial Shape Changes With ATP and Aβ

**FIGURE 1:**
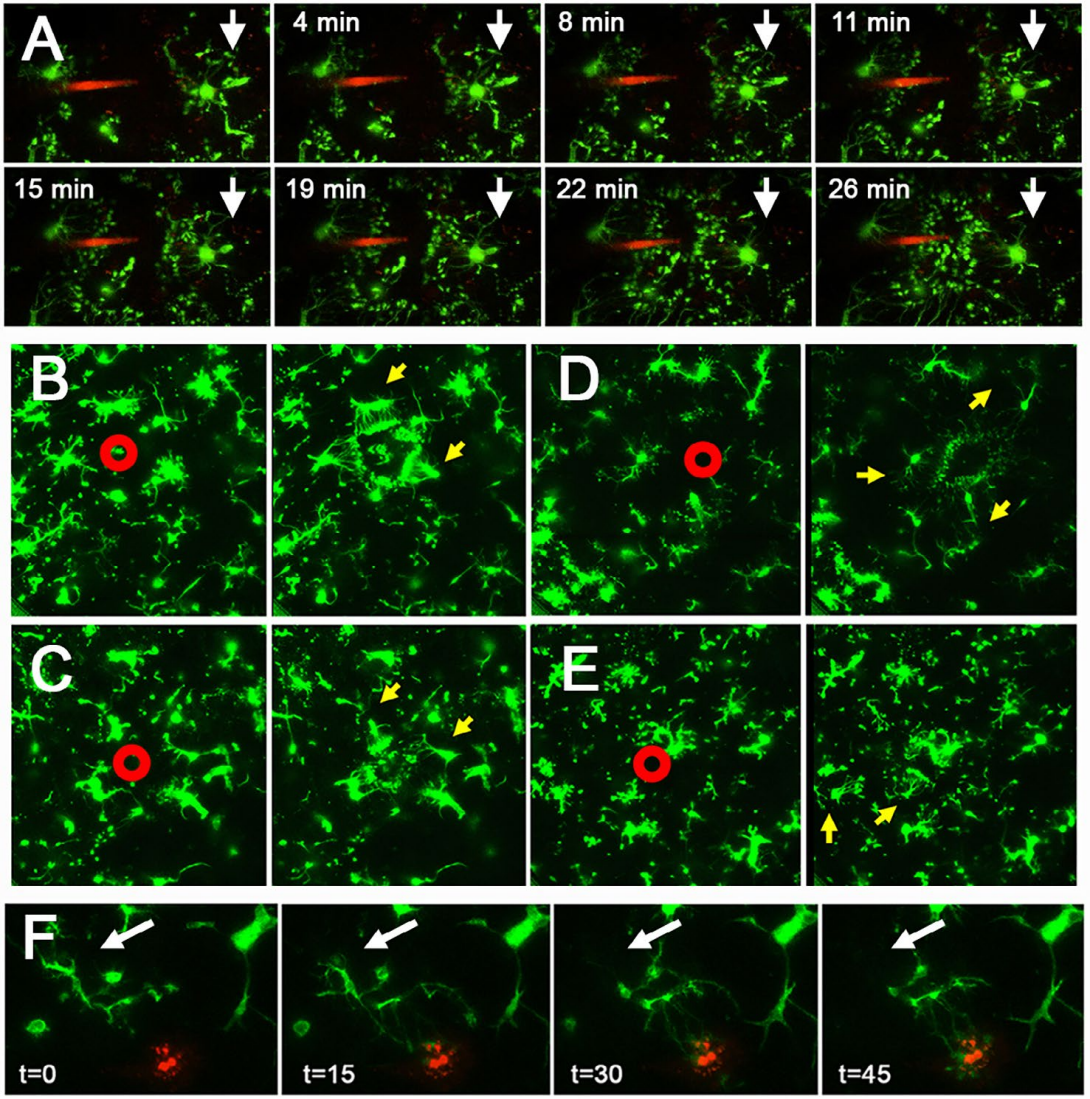
Microglial processes balance extension toward damage with retraction away from damage. Microglia (green) in ex vivo brain tissue extended processes toward a pipet containing 3 mM ATP at the indicated times; retraction of distal processes is indicated with the white arrow (A). Similar results are observed in independent assays of microglia near pipet tips containing 3 mM ATP (indicated by red circles on the left panels) at 20 min (retraction of processes shown by yellow arrows on the right panels, B–E). Fluorescent Aβ42 (red) introduced ex vivo at time 0 resulted in processes extending toward Aβ as well as processes retracting to the microglial cell body (white arrows) between 15 and 45 min (F). Scale bar = 30 μm.

We previously examined how microglial shapes changed in response to acute damage signals ex vivo, using both ATP and Aβ (Sepulveda et al. 2024). Use of a pharmacological inhibitor showed that both stimuli activated microglia by signaling through the homeostatic receptor P2RY12. When ATP was applied through a glass pipette, microglial processes projected toward the pipette within minutes and showed a strong accumulation at the pipette site by 30 min (Sepulveda et al. 2024). In addition to this proximal effect that was noted in earlier studies (Bolmont et al. 2008; Davalos et al. 2005; Haynes et al. 2006; Liu et al. 2023; Nimmerjahn et al. 2005), we made the more novel observation that the microglial processes that were oriented away from the pipette retracted toward the microglial cell body on a similar timescale as the process extensions (Figure 1A, white arrows). This process of dynamic rearrangement was consistently observed across brain slices (Figure 1B–E). In these experiments, the pipette was positioned in close proximity to multiple microglia (insertion sites marked by red circles). After 20 min of ATP diffusion, proximal processes across cells extended toward the pipette, while distal processes retracted (yellow arrows in the adjoining panels, Figure 1B–E). A similar response was observed in the response of microglia to fluorescent Aβ42; over 45 min, the processes near the Aβ42 projected toward the stimulus, while distal processes retracted into the cell body (white arrows, Figure 1F).

### 3.2 Plasma Membrane Differences in Microglial Morphologies

**FIGURE 2:**
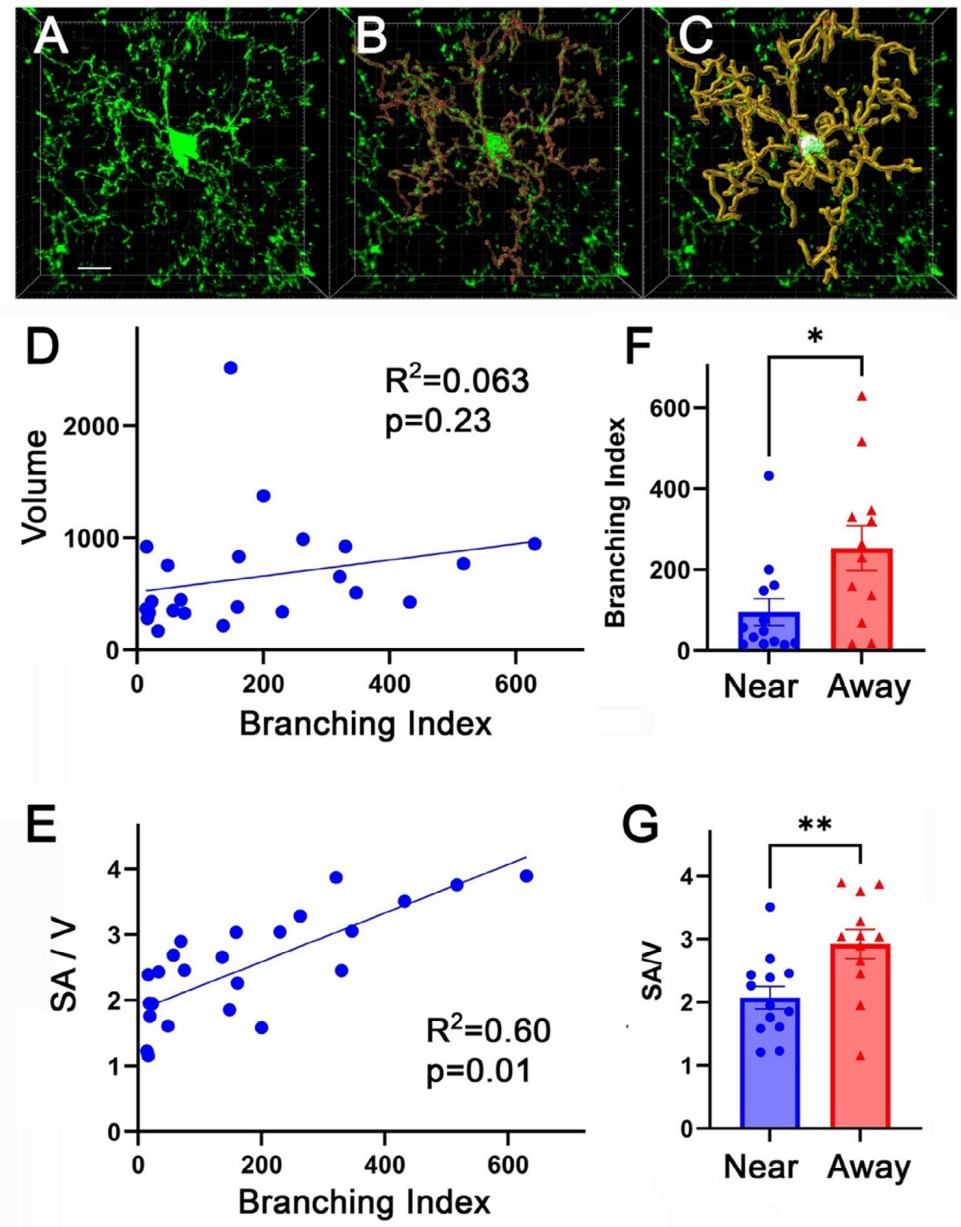
Microglia surface area correlates with cellular complexity. GFP‐labeled microglia in 30 μm brain slices were imaged with confocal microscopy (A). The defined surface area of a single cell is in red (B) and the branching complexity is shown in a stick image of the cellular processes (C). The branching indices were calculated from the stick image and correlated against cellular volumes (D) and the Surface Area to Volume ratios (SA/V) using linear regression (E). The Branching Index and SA/V were statistically compared between cells near a plaque (within 10 μm) or distant from plaques (beyond 100 μm) (F, G); unpaired t tests, *p < 0.05; **p < 0.01. *N* = 25 microglia from four brains of 4‐month‐old *APOE4* ^*+/+*^, CX3CR1‐GFP^+/−^, 5XFAD^+/−^ mice.

Our general hypothesis is that this rearrangement of microglial processes could allow for the conservation of the finite amount of plasma membrane of each cell. Processes extend in one direction and retract from other directions, similar to what occurs during cell migration (Keren 2011). Microglia in vivo display many complex morphologies as they encounter and respond acutely and chronically to brain insults (Paolicelli et al. 2022). We used confocal microscopy to capture and analyze diverse microglial morphologies at different stages of activation. We imaged and analyzed cell shapes of 25 microglia from mouse brains of a model of amyloid accumulation (*APOE4* ^+/+^, 5XFAD^+/−^) and used image analysis software to measure the volume, surface area, and branching complexity of each cell (Figure 2A–C).

The volume of the cell alone was not correlated with the branching index measure of cell complexity (Figure 2D). In contrast, the surface area to volume ratio did correlate strongly with branching index (r^2^ = 0.60, *p* = 0.01) (Figure 2E). Across the microglia we analyzed, the surface area to volume ratio varied approximately three‐fold between the lowest and the highest cells measured (Figure 2E). Thus, cells that had a simpler morphology also had significantly less surface plasma membrane area.

To understand whether chronic amyloid pathology impacted the microglial morphology, we identified amyloid deposits through immunohistochemistry with the MOAB2 antibody. We classified microglia as either being near an amyloid plaque or more distant. As expected, microglia further from plaques were significantly more complex than those near a plaque (*p* < 0.05) (Figure 2F). These distant, more morphologically complex cells exhibited a 50% higher surface area compared to plaque‐proximal microglia (*p* < 0.01) (Figure 2G).

### 3.3 Molecular Analyses of Microglial Activation

**FIGURE 3:**
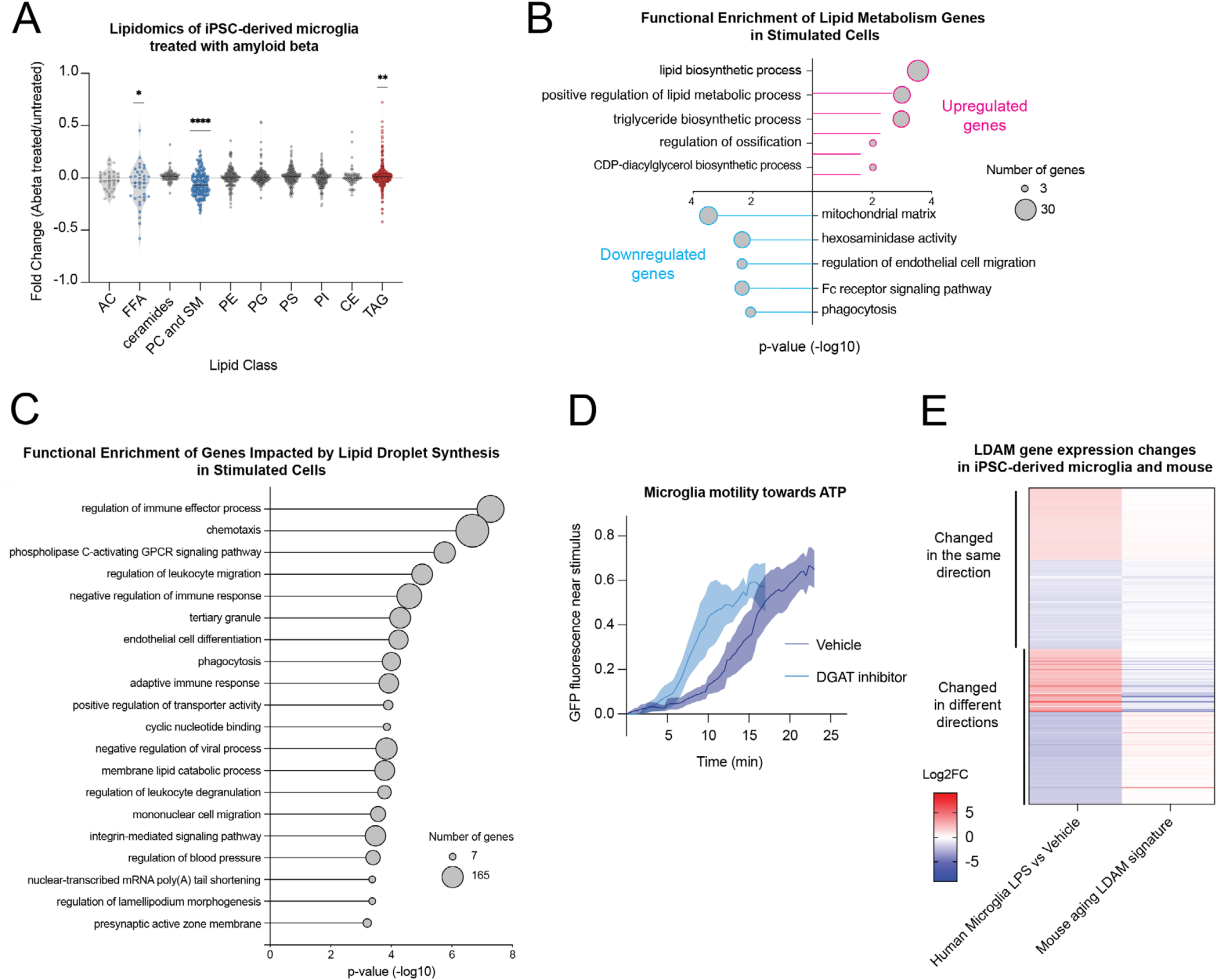
Molecular changes in microglia upon membrane remodeling. (A) Changes in lipids separated by classes in primary mouse microglia treated with amyloid beta. Data replotted from Prakash et al. (2025). One sample t‐test, * *p* < 0.05, **p < 0.01, ****p < 0.0001. (B) Functional enrichment (Zhou et al. 2019) of lipid metabolism gene expression up and downregulated during LPS stimulation of microglia. Transcriptomic data for analysis were drawn from previously published work (Stephenson et al. 2025). (C) Functional enrichment (Zhou et al. 2019) of genes differentially expressed when LPS‐activated human iPSC‐derived microglia are treated with DGAT inhibitors. Transcriptomic data for analysis were drawn from previously published work (Stephenson et al. 2025). (D) Microglia GFP intensity motility toward an area of ATP stimulus in the presence (light blue) and absence (dark blue) of DGAT inhibitors. Data from published work (Stephenson et al. 2025). (E) Heatmap of LDAM gene expression (and expression of human homologues) in LPS‐treated human iPSC‐derived microglia and mouse DGAT inhibition. Data re‐analyzed from published work (Marschallinger et al. 2020; Stephenson et al. 2025).

In our model, any extensive effects of cellular activation on microglial shapes and surface area would require large‐scale lipid remodeling of the plasma membrane, along with transport and storage of excess lipids generated during this remodeling. One possible compartment for this storage in microglia is the lipid droplet (LD), which contains neutral lipids, particularly triacylglycerols (Marschallinger et al. 2020). We hypothesized that triacylglycerols would be synthesized from the remodeled plasma membrane phospholipids in two steps: (1) removal of the phosphate head group from the glycerol backbone, and (2) addition of a third fatty acid side chain. In one test of this hypothesis, we analyzed data from a published study of the lipidomic changes upon activation of primary mouse microglia with Aβ (Prakash et al. 2025). As proposed, the levels of lipids abundant in the plasma membrane—phosphatidylcholine and sphingomyelin—were significantly decreased, and the levels of the lipids stored in the LD, triacylglycerol, were significantly increased (Figure 3A).

We examined whether publicly available transcriptomic data from human iPSC‐derived microglia treated with the extrinsic activation agent lipopolysaccharide (LPS) (Stephenson et al. 2025) reflected increased activity in any lipid biosynthetic pathways. Overrepresentation analysis revealed that LPS activation of microglia resulted in upregulation of lipid biosynthetic processes, particularly the synthesis and shuttling of fatty acids into triacylglycerols (Figure 3B, Tables [Link], [Link]). Using established KEGG pathway lists (https://www.genome.jp/kegg/) and previously published literature on LD in microglia (Haney et al. 2024; Mathiowetz and Olzmann 2024; Olzmann and Carvalho 2019; Stephenson et al. 2025; Victor et al. 2022), we assembled a list of 278 genes involved in membrane and storage lipid metabolism (Table S4). We analyzed their expression patterns during LPS stimulation and in other datasets using Aβ stimulation of iPSC‐derived microglia (Lee et al. 2023; Sun et al. 2023), and LPS stimulation of mouse microglia. We observed upregulation of multiple enzymes spanning triacylglycerol biosynthesis, including the formation of the precursor, diacylglycerol (*GPAT4, AGPAT2,4*); the synthesis of fatty acids (*FASN*) and their activation (*ACSL1,3,4*); and the final step of triacylglycerol synthesis catalyzed by diacylglycerol acyltransferase 2 (*DGAT2*) (Table S5). We also detected increased expression of *CNEP1R1*, which encodes a regulator of the phosphatase lipin (Gao et al. 2024); lipin also generates the triacylglycerol precursor, diacylglycerol (Reue and Wang 2019). In contrast, pathways associated with fatty acid catabolism, including lysosomal and mitochondrial utilization, were downregulated (Table S5). We also observed that activated microglia increased expression of genes *ESYT2, C2CD2L*, and *ANO8*, which govern contact sites between the plasma membrane and the endoplasmic reticulum (a site of LD synthesis (Ralhan et al. 2021)). These changes could facilitate the movement of fatty acids from membrane lipids for storage in triacylglycerols upon cell stimulation, a model described in Section 4.

We also analyzed these existing data to define which transcripts were impacted by inhibiting LD formation through pharmacological inhibition of triacylglycerol synthesis enzymes DGAT1 and DGAT2 (Stephenson et al. 2025). In previous work, we found that microglia treated with DGAT inhibitors displayed altered motility toward an ATP stimulus (Figure 3D), indicating an intimate link between storage lipid synthesis and cell morphology and motility. In LPS‐stimulated cells, inhibition of LD synthesis impacted categories of transcripts related to chemotaxis, cell migration, phagocytosis, membrane lipid catabolism, and membrane structure morphogenesis. In unstimulated cells, we observed that DGAT inhibition caused changes in actin remodeling genes (*ARHGAP/ARGEF*, *RAC2*), and chemotaxis related genes (*CCLs, CCRs, CMKLR1, CNR2, GPR84, ADGRE1/2/3, FPR3*), as well as in genes related to guidance cue signaling (semaphorin genes, *SLIT3*) (Figure 3C, Table S6). These processes of cell migration, phagocytosis, and membrane lipid catabolism could occur independently of each other with microglial shape changes or could be parts of a co‐regulated cell signaling pathway.

There is a subset of lipid droplet associated microglia (LDAM) with high levels of LD, defined initially in aged mouse microglia (Marschallinger et al. 2020). We compared the expression of human homologues of the LDAM gene set in human iPSC‐derived microglia stimulated with LPS to their expression in aged mouse brains (Stephenson et al. 2025). There were 184 transcripts that were significantly changed in the same direction in these two datasets (Figure 3E, Table S8). An enrichment analysis of these genes identified multiple regulators of GTPase activity, cell migration, and cytoskeletal remodeling, such as *DOCK2, ABR, ARHGEF12, RGS10, ARHGAP19, ARHGAP30, RASA4B, MMP14*, and *GPR183* (Table S8). The functional pathways of cell migration and cytoskeleton are necessary for changes in microglial shape during activation (Eyo and Wu 2019; Socodato and Relvas 2024).

Together, these findings demonstrate a shift of gene expression toward triacylglycerol biosynthesis using phospholipid and fatty acid precursors during activation. We suggest that these alterations are indicative of LD synthesis that occurs during cell migration and reduction of plasma membrane areas.

## 4 Discussion

Under resting, homeostatic conditions, microglia have a complex morphology of many fine processes, monitoring the brain microenvironment for signs of damage (Paolicelli et al. 2022). Microglia change shape quickly in response to damage, responding to laser ablation of cells (Liu et al. 2023; Nimmerjahn et al. 2005), to ATP (Davalos et al. 2005; Sepulveda et al. 2024), and to Aβ (Bolmont et al. 2008; Gyoneva et al. 2016; Liu et al. 2023; Sepulveda et al. 2024). With age (Damani et al. 2011; Sepulveda et al. 2024) and *APOE4* genotype (Fitz et al. 2021; Liu et al. 2023; Sepulveda et al. 2024; Yin et al. 2023), these responses slow. The projection of processes toward sites of damage is accompanied by the retraction of processes away from the site of damage (Figure 1). These changes are consistent with the fast redistribution of plasma membrane from one region of the microglia to another.

Over a longer time, microglia undergo dramatic morphological changes, taking on shapes that have fewer and thicker processes or have simpler, more rounded shapes (Reddaway et al. 2023; Vidal‐Itriago et al. 2022). In the AD brain, microglia that accumulate in the vicinity of amyloid plaques (Griffin et al. 1989) adopt an amoeboid shape (Stalder et al. 1999) and show decreased homeostatic gene expression (Paasila et al. 2019). In the 5XFAD model of brain amyloid, microglia near plaques had a greater amoeboid morphology while those further from plaques had more extended processes (Prakash et al. 2025). The number of microglial LD (as defined by neutral lipid staining and the presence of PLIN2) was highest in the regions of the 5XFAD brain with extensive plaque pathology (i.e., the subiculum) (Prakash et al. 2025). More specifically, microglia containing LDs were enriched in the regions within 10 μm of amyloid plaques, with LD load decreasing with increasing distance from the plaques; these findings were confirmed in human brain sections (Prakash et al. 2025).

**FIGURE 4:**
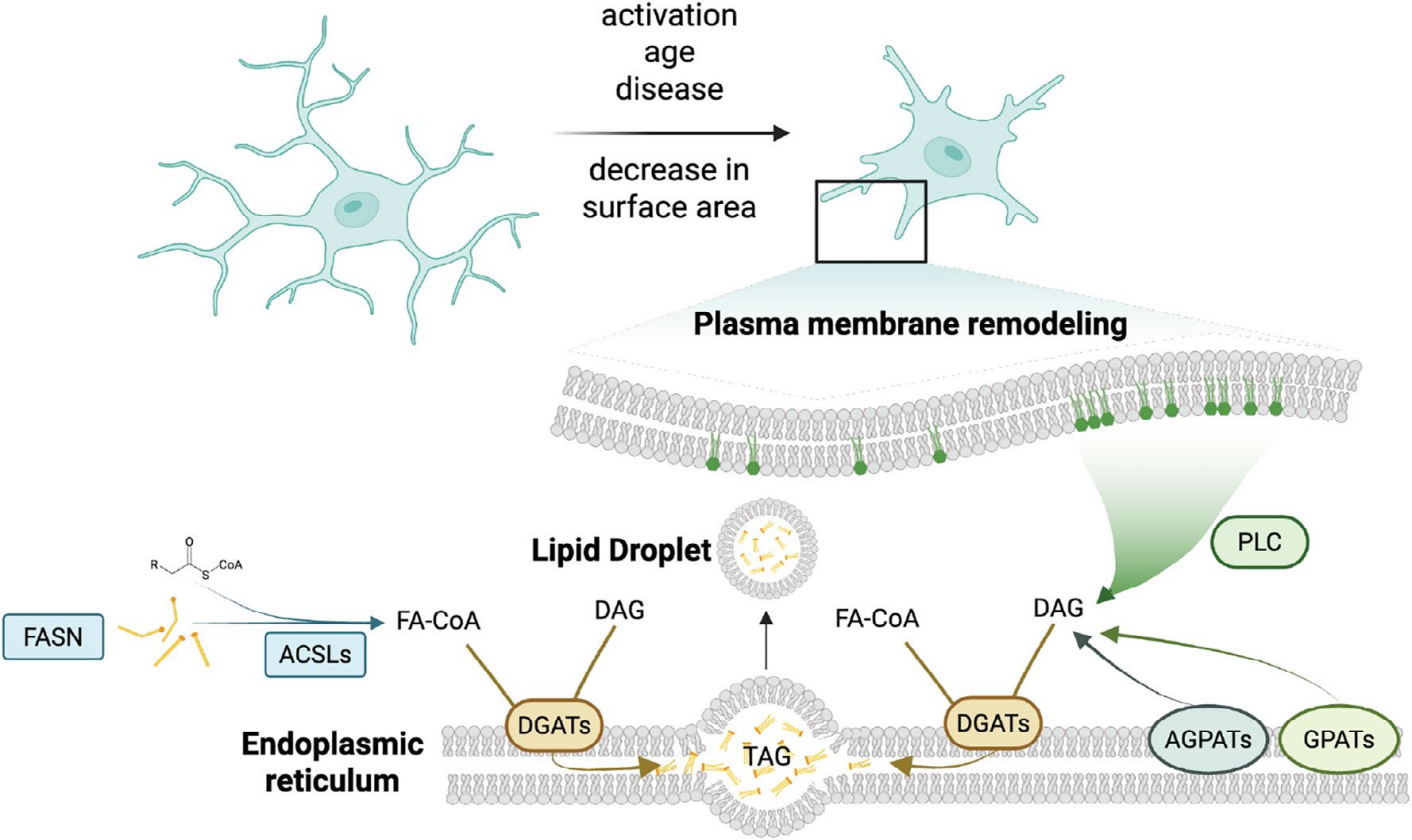
Model of microglial membrane remodeling. A schematic showing links between microglial activation, membrane remodeling, and lipid storage in lipid droplets. Figure created using BioRender.

These changes to microglial shapes with stimulation and activation must involve substantial remodeling of the plasma membrane. This lipid bilayer could be redistributed rapidly during acute process motility (Figure 1) or relocated more extensively during chronic shape changes (Figure 2). Activated microglia have increased LD (Haney et al. 2024; Stephenson et al. 2025), which also exist in chronic paradigms like the 5XFAD amyloid mouse model (Marschallinger et al. 2020; Prakash et al. 2025). In our study of 5XFAD brains, microglia near amyloid plaques had less surface area compared to microglia more distant from plaques (Figure 2). This novel measurement of a basic cellular property of microglial cells is consistent with observations of amoeboid phagocytic cells in liver (Kuppfer cells), which also showed about half the surface area of ramified microglia (Lawson et al. 1990). We propose that LD formation provides a dynamic mechanism for storing excess lipids that are removed from the plasma membrane as part of the reduction in surface area (Figure 4). These LD could remain at high levels under chronic activation, such as around amyloid plaques, to allow flexibility of microglial process removal, extension, and recovery.

Our analysis of existing datasets shows that activated microglia upregulate genes important to triacylglycerol synthesis (Stephenson et al. 2025) including the rate‐limiting enzyme *DGAT2*, the enzymes producing precursors (*AGPAT*s, *GPAT*s), and the enzymes involved in fatty acid generation and acylation (*FASN*, *ACSL*s) (Table S4). To determine whether these lipid gene changes in LPS‐stimulated iPSC‐derived microglia were recapitulated in other contexts, we analyzed two additional datasets: one where iPSC‐derived microglia were treated with Aβ (Sun et al. 2023) and another where mice expressing human *APOE3* or *APOE4* alleles were treated with LPS (Lee et al. 2023). In both, there was increased expression of fatty acid acylation enzymes (*ACSL1–4*), triacylglycerol precursors (*AGPAT4*, *PLC*), and LD‐associated proteins (*PLIN3*). Activation of mouse microglia with Aβ reduced plasma membrane species (phosphatidylcholine and sphingomyelin) while increasing triacylglycerol (Stephenson et al. 2025). These findings suggest a metabolic reprogramming toward lipid storage in human and mouse cells and tissue that correlates with microglia activation. These changes align with reports of increased LD that accumulate in *APOE4*, aged, and diseased microglia (Haney et al. 2024; Lee et al. 2023; Tcw et al. 2022).

We also noted downregulation of mitochondrial and lysosomal pathways involved in lipid degradation, suggesting that microglial LDs may not be primarily destined for energy production. We suggest that they may act, in part, as reservoirs for excess membrane lipids, available for later mobilization. In support of this conclusion, we found increased expression of phospholipid lipases (*PNPLA6*, *PLC*) and membrane‐remodeling genes (*ARF1*, *VMP1*), consistent with active plasma membrane reorganization alongside neutral lipid accumulation. Ultrastructural electron microscopy images from aged mice (Marschallinger et al. 2020) as well as iPSC‐derived microglia (Haney et al. 2024) showed proximity of the plasma membrane and membrane‐derived endo‐lysosomal compartments to LD. We also found upregulation of transcripts related to contact sites between the plasma membrane and endoplasmic reticulum (Sun et al. 2019), suggesting that the LD biogenesis likely involves both surface and internal plasma membranes.

In addition to increased intracellular lipid storage, excess plasma membrane lipids during membrane remodeling could also be released from the microglia through the process of lipid efflux. Lipid efflux would require lipid transporters such as ABCG1 and ABCA1, as well as an apolipoprotein acceptor such as apoE or apoJ (Kim et al. 2008). The apoE4 protein is less efficient for lipid efflux compared to apoE3 or apoE2 (Michikawa et al. 2000), which may limit removal of excess lipids from microglia under activated conditions. This deficit could contribute to some of the higher LD accumulation in *APOE4* cells (Farmer et al. 2019; Haney et al. 2024; Stephenson et al. 2025). Alternatively, membrane lipids could be released via exosomes, a process promoted in macrophages by activation of the purinergic receptor P2X7R (Gulinelli et al. 2012). Concomitant activation of phospholipid internalization and efflux could allow the necessary reorganization of both the inner and outer membrane leaflets during plasma membrane reductions.

Transcriptomic analyses have identified many classes of microglia, with speculation about their functions (Dolan et al. 2023; Green et al. 2024; Olah et al. 2018; Sun et al. 2023). Several seem to be homeostatic in nature with high levels of the P2RY12, the receptor which mediates some of the acute microglial shape changes in this study. There are several classes of microglia in various neurodegenerative conditions (Friedman et al. 2018; Keren‐Shaul et al. 2017; Krasemann et al. 2017; Mathys et al. 2017). Our model would suggest that the LD‐laden microglia (Haney et al. 2024; Marschallinger et al. 2020) are formed due to previous membrane reorganizations in response to activating stimuli such as amyloid in mice (Prakash et al. 2025) and humans (Haney et al. 2024).

Tests of these hypotheses could focus on defining causal connections between microglial membrane changes and LD accumulation. One approach could make use of conditional, microglial‐specific knockouts of genes related to the pathways described here for the synthesis of triacylglycerol (e.g., DGAT, PLC). Another approach could explore the effect of promoting lipid efflux on the abundance of amyloid plaque‐proximal LD. In addition, in vitro assays using fluorescently traceable lipid precursors could allow direct visualization of whether activation‐induced redistribution of lipids occurs between membranes and LD.

There is speculation about the roles of LD in many cell types, including use as energy sources, collection of lipid debris or plasma membranes after phagocytosis, and sources of lipid for delivery to other cells (Goodman et al. 2024; Qi et al. 2021; Ralhan et al. 2023). Our findings also position LD formation as a result of microglial membrane remodeling, occurring when there are reductions in cell surface area in the formation of more amoeboid shapes. By linking rapid redistribution of plasma membrane lipids to longer‐term sequestration, microglia could adapt to both acute and chronic stimuli. Disruption of this balance, as seen in aging, *APOE4* genotype, and neurodegenerative disease, may impair the cellular plasticity that is essential for microglial function. Regulation of lipid mobilization and storage could thus offer novel ways to modulate microglial function.

## Author Contributions

G.W.R., J.S., and P.S.N. developed the ideas and designed the approach of the work. J.S. conducted the ex vivo experiments. G.S. and G.S.H. developed assays and conducted analyses of microglial shapes. G.W.R. and P.S.N. analyzed and interpreted transcriptomic data. G.W.R. and P.S.N. wrote the manuscript and all authors generated figures and provided edits. The contributions of the NIH author (P.S.N.) were made as part of their official duties as NIH federal employees, are in compliance with agency policy requirements, and are considered Works of the United States Government. However, the findings and conclusions presented in this paper are those of the authors and do not necessarily reflect the views of the NIH or the U.S. Department of Health and Human Services. All authors reviewed and approved of the manuscript.

## Funding

This work was supported by the Albert and Linda Rosecan Charitable Foundation and the Georgetown University Medical Center. There was support from National Institutes of Health (NIH) extramural grants F99 NS134164 (J.S.) and T32 GM144880 (G.S.). This research was also supported by the Intramural Research Programs of the National Institute of Diabetes and Digestive and Kidney Diseases (NIDDK) (1ZIADK075158) within the National Institutes of Health (NIH).

## Conflicts of Interest

The authors declare no conflicts of interest.

## Supporting information


**Table S1:** Fold changes and FDR‐corrected *p* values for gene expression of microglia with and without LPS treatment.

**Table S2:** GO terms for differentially upregulated lipid genes upon LPS treatment compared to vehicle in APOE3 microglia.

**Table S3:** GO terms for differentially downregulated lipid genes upon LPS treatment compared to vehicle in APOE3 microglia.

**Table S4:** Curated lipid metabolism genes for membrane and storage lipids from KEGG pathways and literature.

**Table S5:** Comparative data across three datasets for lipid genes differentially expressed upon LPS treatment.

**Table S6:** Fold changes and FDR‐corrected *p* values for fold change gene expression under DGAT inhibitor treatment compared to vehicle and LPS‐treated controls.

**Table S7:** GO terms for differentially expressed genes upon DGAT inhibitor treatment compared to vehicle and LPS‐treated controls.

**Table S8:** Human homologues of “LDAM” gene list (Marschallinger et al. 2020), fold changes and FDR‐corrected *p* values for gene expression of microglia with and without LPS, treatment and enrichment of GO categories for genes regulated in the same and opposite directions in aged mouse microglia and human iPSC‐derived microglia datasets.

## Data Availability

The data that support the findings of this study are available from the author first (G.W.R.) or last (P.S.N.) upon reasonable request.
